# Reducing Antibiotic Prescription Errors in the Emergency Department: A Quality Improvement Initiative

**DOI:** 10.1097/pq9.0000000000000314

**Published:** 2020-06-26

**Authors:** Kathryn E. Kasmire, Crista Cerrone, Eric C. Hoppa

**Affiliations:** From the * Division of Emergency Medicine, Penn State Health Milton S. Hershey Medical Center, Penn State College of Medicine, Hershey, PA; † Division of Emergency Medicine, Nationwide Children’s Hospital, The Ohio State University College of Medicine, Columbus, OH; ‡ Division of Pediatric Emergency Medicine, Connecticut Children’s University of Connecticut School of Medicine, Hartford, CT.

## Abstract

**Introduction:**

Discharge prescription errors from the pediatric emergency department (ED) are common. Despite the implementation of clinical pathways for common infections recommending specific antibiotic therapy and aids built into the electronic health record, errors in antibiotic prescriptions for patients discharged home from the ED persist.

**Methods:**

We developed and implemented ED antibiotic discharge order panels for urinary tract infection (UTI) and skin and soft tissue infections (SSTI) that modeled antibiotic therapy from our institutional clinical pathways. We aimed to reduce antibiotic prescription errors by 50% within 6 months of implementation.

**Results:**

With the implementation of the ED discharge order panels, the overall error rate for prescriptions for UTI and SSTI improved from a baseline rate of 29.3% to 12.6% (*P* < 0.001). Individually, the baseline number of prescriptions with errors for UTI and SSTI improved from 26.1% and 32.8%, respectively, to 13.8% and 12.5% within 6 months. Sustained improvement continued for 17 months after the implementation of the order panels.

**Conclusions:**

Development and implementation of ED antibiotic discharge order panels decrease antibiotic prescription errors for UTI and SSTI by improving compliance with institutional clinical pathways. Additional order panels should be developed and implemented for other conditions to help reduce discharge prescription errors.

## Abstract 2

Supplemental Digital Content is available in the text.

## INTRODUCTION

Antibiotics are frequently prescribed in the emergency department (ED) for common pediatric infections, including urinary tract infections (UTI) and skin and soft tissue infections (SSTI). Prescription errors are common both at our institution and in other centers.^1,2^ ED prescription errors are common in academic centers and may be more common in pediatric patients than in adult patients.^1^ One strategy that has reduced rates of prescription errors in the pediatric ED is an automated alert system built into the electronic health record (EHR).^3^ Despite these safeguards built into the EHR, prescription errors still occur.^4,5^ Challenges in prescribing antibiotics in pediatric patients include weight-based dosing, variable dosing, duration of therapy, and dosing intervals for different indications. The choice of correct liquid or tablet formulation depends on the child’s ability to take tablets and the conversion of doses into volume for liquid formulations. Dosing errors are the most common type of prescription error in pediatrics.^6^ In academic institutions, prescriptions are often written by rotating residents who may have varying experience with pediatric prescriptions leading to increased risk of errors. Strategies across pediatric disciplines that have reduced medication errors include the use of e-prescriptions, electronic ordering with dosing recommendations, and indication-specific prescribing.^7–11^ Kadmon et al^10^ reported that despite the initial reduction of inpatient prescription errors with the implementation of computer order entry, increasing inpatient prescription error rates can return over time, with new medications in the order system and new physicians. Updating and revising clinical support tools can decrease these error rates.^12^

Clinical pathways help to standardize care for common ED diagnoses and improve overall timely care.^13–17^ They can help guide both ED management and discharge care, including outpatient prescriptions. Clinical pathways for common pediatric infections often recommend the selection of appropriate antibiotics for outpatient care.^18^ Antibiotic selection to treat common pediatric infections, including UTI and SSTI, should be guided by local antibiotic resistance patterns and focus on the use of narrow-spectrum antibiotics. Poole et al^18^ demonstrated that a clinical pathway for UTI improved narrow-spectrum prescribing for UTIs discharged from the ED and urgent care settings.

Our ED uses a robust library of clinical pathways to guide the evaluation and management of common pediatric infections, including UTI, SSTI, community-acquired pneumonia, animal bite, and preseptal cellulitis. Each pathway has recommendations for outpatient therapy guided by the antibiotic stewardship program using local resistance patterns. The recommendations include appropriate antibiotic guidance (including alternatives for patients with allergies), weight-based dosing, dosing interval, and duration of therapy. Despite having these pathways in place, deviation from prescription recommendations and prescription errors are still common.

We developed a multidisciplinary quality improvement initiative to reduce antibiotic prescription errors in the ED through the implementation of diagnosis-specific standardized discharge prescription order panels. The team created and implemented order panels modeling antibiotic recommendations of our UTI and SSTI clinical pathways. The SMART (Specific, Measurable, Achievable, Realistic, and Timely) aim was to reduce ED antibiotic prescriptions with errors for UTI and SSTI by 50% within 6 months of implementation. The intervention was a high-reliability intervention anticipated to produce sustainable improvement.

## METHODS

### Context

We conducted the project at an academic urban tertiary children’s hospital with an annual ED volume of 60,000 patients. The ED is staffed by board-certified pediatric emergency medicine attending physicians, pediatric emergency medicine fellows, pediatric, emergency medicine, and family medicine residents, physician assistants, and nurse practitioners. The institution has a robust clinical pathway program with many pathways involving both ED and inpatient care (https://www.connecticutchildrens.org/clinical-pathways/). The hospital clinical effectiveness committee reviews and approves all clinical pathways before implementation. The monitoring of quality metrics for the pathways occurs monthly. Each pathway undergoes an annual quality review, a triennial review with a full pertinent medical literature review, and pathway revision as necessary. Our institutional antibiotic stewardship committee guides antibiotic selection and recommendations for dosing and length of the antibiotic course for the clinical pathways.

### Intervention

**Fig. 1.**
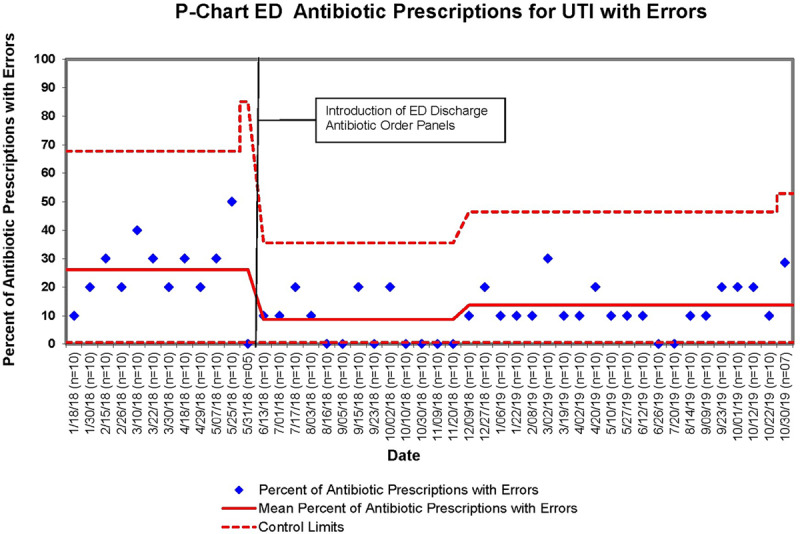
P-chart for ED antibiotic prescriptions for UTI with errors.

**Fig. 2.**
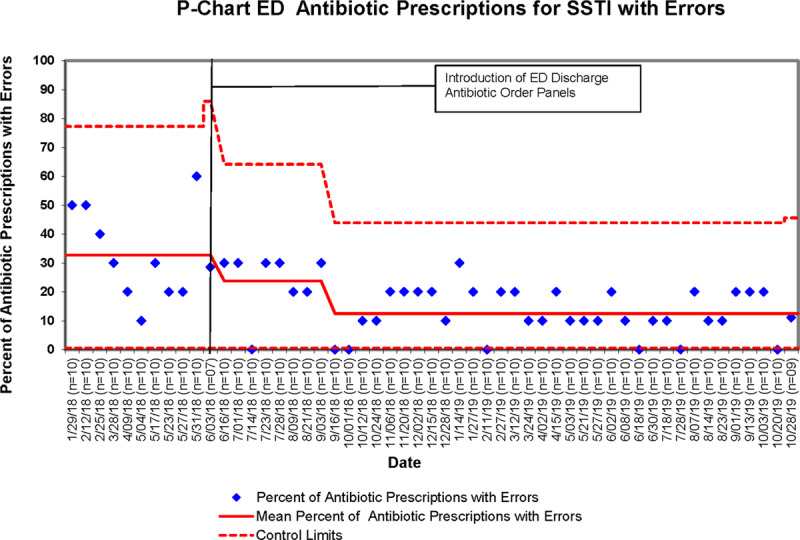
P-chart for ED antibiotic prescriptions for SSTI with errors.

A multidisciplinary team including a pediatric emergency medicine attending physician, pediatric emergency medicine fellow, pediatric resident, quality data analyst, and information technology analyst met to design diagnosis-specific ED discharge antibiotic order panels to model the outpatient antibiotic recommendations of preexisting clinical pathways for UTI and SSTI (**Fig. 1, Supplemental Digital Content 2**, UTI Clinical Pathway, available at http://links.lww.com/PQ9/A191 and **Fig. 2, Supplemental Digital Content 2**, SSTI Clinical Pathway, available at http://links.lww.com/PQ9/A192) (Table 1). The order panels included the correct antibiotic (including alternatives for patients with allergies), dose (including maximum dose), dosing interval, and duration of therapy. The prescriber could then choose the appropriate formulation of antibiotic (tablet versus liquid) based on the patient’s age and ability to swallow pills. The order panels were created in the EHR and accessed by entering a specific diagnosis (ie, UTI, cellulitis, abscess) or specific antibiotic (ie, cephalexin, clindamycin) into the prescription order panel on the patient discharge navigator. The panel automatically populated the correct antibiotic, dose, and dosing interval for the discharge prescription.

**Table 1.**
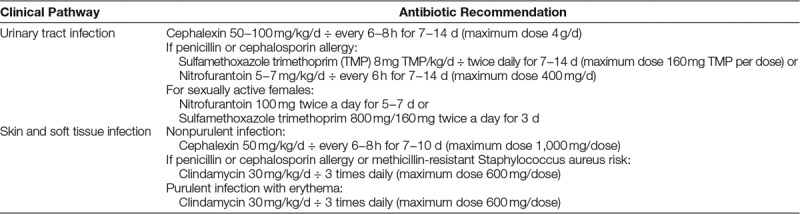
Antibiotic Recommendations for Treatment of UTI and SSTI in Institutional Clinical Pathways

The improvement team used the plan-do-study-act cycles described below to create and implement the order panels. Our information technology analyst created the first draft of the order panels with input from all improvement team members. The ED attending and resident physician trialed the order panels in the testing environment of the EHR. Feedback from initial testing led to changes in dose calculations to model the clinical pathways and the revision of the order panels allowing access to them by entering a specific diagnosis (ie, UTI, cellulitis, or abscess). After these changes, the team shared the order panels with a variety of end users, allowing them to use the order panels in the testing area of the EHR. Based on feedback from these sessions, there was no need for additional revisions. Education for end users on the use of the order panels occurred before final implementation. The improvement team created a document with step-by-step instructions for accessing and using the order panels. Vetting of the document by physician members of the improvement team and a small group of end users led to revisions of the document to clarify the process for access and use of the order panels. This document was disseminated by e-mail to all pediatric emergency medicine attending physicians, fellows, and ED advanced practice providers, as well as residents rotating through the ED. The pediatric emergency medicine attending and the fellow on the improvement team introduced the order panels to ED staff during the monthly ED meeting in the month before final implementation.

### Measures

We extracted data from an existing clinical pathway dataset to track pathway utilization and specific quality metrics. All patients with International Classification of Diseases, Tenth Revision (ICD 10) diagnostic codes for UTI and SSTI, who met the criteria for the respective pathway, were included in the quality dataset (Table 2). We only included patients who were discharged home from the ED in the analysis. We defined prescription errors as deviations from antibiotic recommendations in the respective clinical pathway. We included (1) use of an antibiotic not recommended by the clinical pathway, (2) incorrect antibiotic dose (mg/kg), (3) incorrect dosing interval, or (4) incorrect duration of antibiotic therapy. We recorded both the number of patients with an error and the total number of errors. All errors had a manual ED chart review to determine if there was a reason for the deviation from the clinical pathway (ie, past bacterial culture results or other patient-specific factors). If the prescription had clinically justified deviations, we did not count it as an error.

**Table 2.**
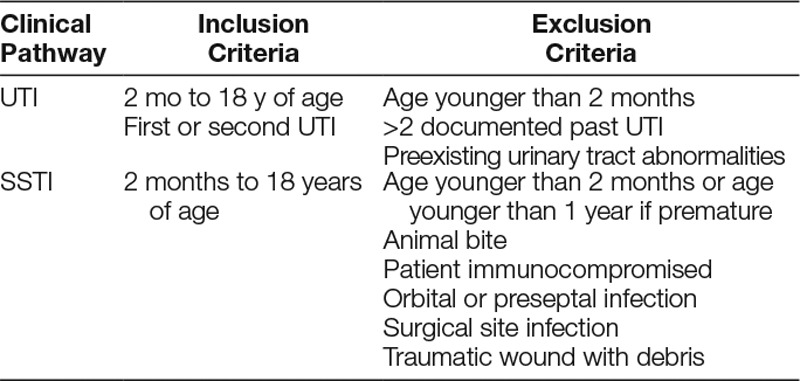
Inclusion and Exclusion Criteria for the UTI and SSTI Pathway

Baseline percentage of prescriptions with errors were reviewed for 5 months from January 2018 through May 2018 before order panel implementation. We introduced the discharge antibiotic order panels for use in the ED on June 4, 2018. Data after implementation were reviewed monthly from the clinical pathway dataset in the same manner as the baseline data. Overall, we tracked 17 months of data after the implementation of the order panels until October 31, 2019.

### Analysis

Given the week-to-week and month-to-month variations in the number of cases of UTI and SSTI, which met clinical pathway criteria, we grouped cases in sets of 10 for data analysis. Data were plotted and tracked for both UTI and SSTI on statistical process control charts. Mean shifts were plotted after 8 consecutive data points above or below the mean with an identifiable cause, signaling special cause variation.^19^

### Ethical Considerations

This project was deemed to be a quality improvement project and not human subjects research. Therefore, the institutional review board did not require review and approval.

## RESULTS

The baseline mean rates of discharge antibiotic prescriptions with errors for UTI and SSTI were 26.1% and 32.8%, respectively. After the implementation of discharge antibiotic order panels, the mean percentage of UTI prescriptions with errors initially decreased to 8.8% (Fig. 1). The mean regressed to 13.8% in December 2018 and sustained at this level for 11 months through October 2019. The sustained reduction in errors represented a 47.1% decrease in prescriptions with errors. Mean discharge prescriptions for SSTI with errors initially decreased to 23.8% and, over time, improved to 12.5% (Fig. 2). The project demonstrated immediate improvement within 6 months of implementation that continued for a total of 17 months. Overall the error rate improved by 61.8% for prescriptions for SSTI. Overall the project decreased prescription errors from a baseline of 29.3% to 12.6% (*P* < 0.001) for both diagnoses combined (Table 3).

**Table 3.**
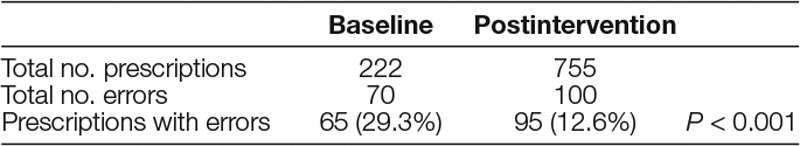
Prescription Errors Before and After Implementation of ED Antibiotic Discharge Order Panels

**Fig. 3.**
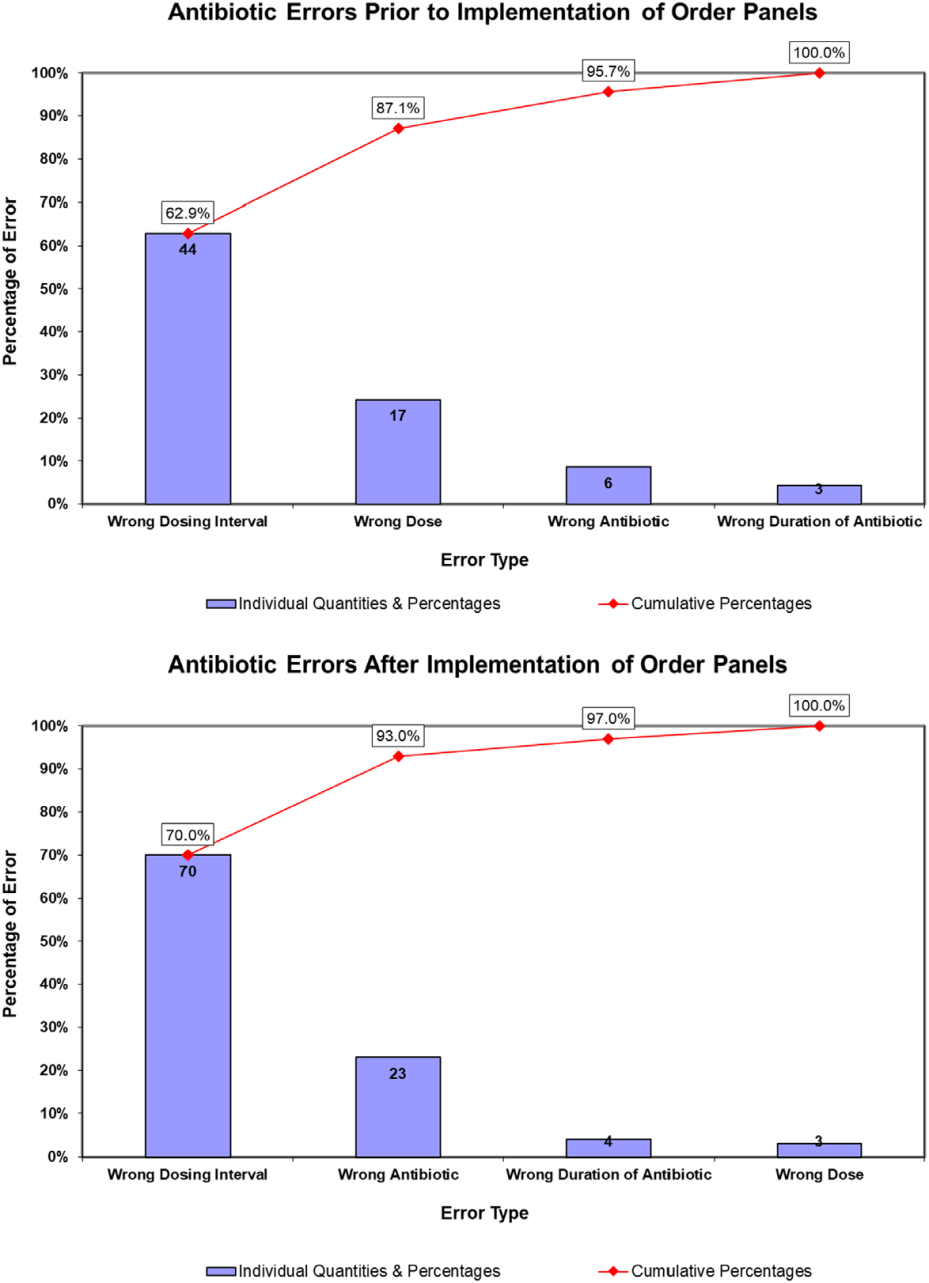
Pareto charts comparing baseline prescription errors to errors after implementation of order panels.

By far, the most common type of error was the wrong antibiotic dosing interval, both pre- and postimplementation of the order panels (Fig. 3). The wrong antibiotic dose was the second most common error before the project, whereas wrong antibiotic selection was the second most common after implementation of the order panels.

## DISCUSSION

The development and implementation of antibiotic discharge order panels for UTI and SSTI decreased prescription errors by improving compliance with institutional clinical pathways. The overall reduction in prescriptions with errors 6 months after the implementation of the order panel for UTI and SSTI was 47.1% and 61.8%, respectively. These results nearly met our goal of a 50% reduction in errors for UTI and surpassed the goal for SSTI.

Clinical pathways provide standardized, evidence-based care. Any intervention that can increase compliance with such pathways eliminates unnecessary variation in the treatment of common conditions. Antibiotic therapy recommended based on local susceptibility patterns of common bacterial pathogens is a crucial tenant of antibiotic stewardship. Several past studies document the use of technology and the EHR to improve antibiotic stewardship.^20–22^ Implementation of our discharge antibiotic order panels accomplished improvement both in the realm of standardizing care and promoting the use of narrow-spectrum antibiotics and appropriate duration of therapy.

Although the implementation of order panels achieved a significant reduction in antibiotic prescription errors, the antibiotic dosing interval remained the most common error after implementation. There was also an increase in the percentage of wrong antibiotic selection errors based on the clinical pathway. These persistent errors in antibiotic dosing interval and the higher rate of incorrect antibiotic use after order panel implementation likely reflect a population of ED providers who did not use the order panels. The majority of the dosing interval errors were from twice-daily dosing prescriptions for cephalexin for both UTI and SSTI instead of the recommended dosing of 3 or 4 times a day. These errors likely resulted from ED providers using other dosing references that suggest a dosing interval of twice daily for cephalexin.^23^ Despite these references, we considered this an error as it did not align with our clinical pathways and the most current evidence-based recommendations for the treatment of UTI and SSTI.^24,25^

The current configuration of our EHR made it impossible to track order panel use in the EHR. The final percentages of UTI and SSTI antibiotic prescriptions with errors, 13.8% and 12.5%, respectively, demonstrate less than 100% provider usage of the order panels 17 months after implementation. Given the level 1 reliability of the interventions of both the order panels themselves and the educational initiatives around them, one would expect an 80%−90% adoption of order panel use, which is reflected in the results of our interventions. A higher reliability intervention such as a hard stop in the EHR mandating the use of the order panel may help to increase panel use and decrease the error rate even further. Future improvement work should center on such an intervention.

Our improvement was immediate after implementation and sustained over 17 months. Improvements seen initially with UTI prescription errors regressed somewhat after initial improvement but remained well above the preimplementation baseline for 11 months. Factors leading to immediate improvement included ease of use of the refined and pretested order panels, initial educational initiatives, and provider buy-in. The increase in UTI prescriptions with errors 7 months after implementation most likely occurred as the impact of the initial ED provider education waned. Sustained improvement continued because of ongoing monthly education to rotating residents and ongoing provider buy-in. Larger-scale reeducation initiatives may have led to an ongoing reduction in prescription errors.

### Limitations

This project tracked the percentage of prescriptions with errors after the implementation of antibiotic order panels. However, as previously noted, it was unable to monitor for actual use of the order panels. There was a significant improvement in error rates, but we are unable to demonstrate that the implementation of the discharge order panels directly led to all of the improvement. With the project, there may have been an observer effect where provider awareness about the project leads to increased vigilance of following the institutional pathways without the use of the order panels. This possibility could be especially true given the immediate improvement after implementation but would be unlikely to explain the sustained improvement. Over time, with the introduction of more clinical pathways, providers may have been more aware of the institutional pathways and more apt to follow the pathway recommendations without explicitly using the discharge order panels. In the future, establishing a way to directly monitor the use of the ED order panels within the EHR could better quantify their effect.

Another limitation of our project could have been patient-level factors that lead to deviation in therapy from our clinical pathways for UTI and SSTI. Though the improvement team conducted a chart review for every patient with a prescription error, there may have been factors not documented in the chart, which would have led to deviation from the clinical pathway, therefore, increasing the number of prescriptions with errors. This circumstance may explain some of the incorrect antibiotic selection and antibiotic duration but would be less likely to explain antibiotic dosing and frequency errors.

A final limitation of this project is that we evaluated a process measure of prescription errors and not a specific outcome measure such as clinical improvement or treatment failure and subsequent need for hospitalization. We also only know what antibiotic was prescribed, and given the pathway recommendations for more frequent dosing of medications such as cephalexin, we do not know if the patient filled their prescription or if they completed the antibiotic as prescribed, including the appropriate dosing interval and duration of therapy.

### Next Steps

The next steps of this project should focus on further integrating the current order panels into the ED system and expanding the order panels to other domains. Further integration of the discharge order panels into the EHR, such as integrated prompts suggesting their use based on discharge diagnosis, would likely lead to increased provider use of the order panels and a further improvement in prescription errors.

Expanding the discharge order panels to other clinical pathways that incorporate outpatient antibiotic management may lead to improved compliance with local pathways or consensus recommendations. Also, this work could be expanded from the ED to the inpatient arena for patients discharged home requiring additional oral antibiotic therapy. This work can also serve as a model for other pathways or clinical conditions where specific outpatient prescription therapy outside of antibiotics is required and would benefit from standardization.

## CONCLUSIONS

With the implementation of discharge antibiotic order panels, the percentage of prescriptions with errors for UTI and SSTI improved by 47.1% and 61.8%, respectively, by increasing compliance with institutional clinical pathways. The initial improvement was sustained for 17 months following the order panel implementation. Future work should concentrate on expanding these panels to other pathways and conditions. Also, higher reliability interventions, including EHR prompts to use the order panels, could sustain and increase the scope of the improvement initiative.

## DISCLOSURE

The authors have no financial interest to declare in relation to the content of this article..

## ACKNOWLEDGMENTS

Danny Vo assisted with the development of the order panels studied in this project.

## Supplementary Material



## References

[1] Murray KA Belanger A Devine LT Emergency department discharge prescription errors in an academic medical center. Proc (Bayl Univ Med Cent). 2017; 30:143–146 28405061 10.1080/08998280.2017.11929562 PMC5349807

[2] Rinke ML Moon M Clark JS Prescribing errors in a pediatric emergency department. Pediatr Emerg Care. 2008; 24:1–8 18165799 10.1097/pec.0b013e31815f6f6c

[3] Sethuraman U Kannikeswaran N Murray KP Prescription errors before and after introduction of electronic medication alert system in a pediatric emergency department. Acad Emerg Med. 2015; 22:714–719 25998704 10.1111/acem.12678

[4] Nelson CE Selbst SM Electronic prescription writing errors in the pediatric emergency department. Pediatr Emerg Care. 2015; 31:368–372 25931343 10.1097/PEC.0000000000000428

[5] Caruso MC Gittelman MA Widecan ML Pediatric emergency department discharge prescriptions requiring pharmacy clarification. Pediatr Emerg Care. 2015; 31:403–408 25996232 10.1097/PEC.0000000000000457

[6] Wong IC Ghaleb MA Franklin BD Incidence and nature of dosing errors in paediatric medications: a systematic review. Drug Saf. 2004; 27:661–670 15230647 10.2165/00002018-200427090-00004

[7] Galanter W Falck S Burns M Indication-based prescribing prevents wrong-patient medication errors in computerized provider order entry (CPOE). J Am Med Inform Assoc. 2013; 20:477–481 23396543 10.1136/amiajnl-2012-001555 PMC3628069

[8] Johnson KB Lehmann CU; Council on Clinical Information Technology of the American Academy of Pediatrics. Electronic prescribing in pediatrics: toward safer and more effective medication management. Pediatrics. 2013; 131:e1350–e1356 23530183 10.1542/peds.2013-0193 PMC8194477

[9] Ginzburg R Barr WB Harris M Effect of a weight-based prescribing method within an electronic health record on prescribing errors. Am J Health Syst Pharm. 2009; 66:2037–2041 19890088 10.2146/ajhp080331

[10] Kadmon G Bron-Harlev E Nahum E Computerized order entry with limited decision support to prevent prescription errors in a PICU. Pediatrics. 2009; 124:935–940 19706588 10.1542/peds.2008-2737

[11] Kaushal R Kern LM Barrón Y Electronic prescribing improves medication safety in community-based office practices. J Gen Intern Med. 2010; 25:530–536 20186499 10.1007/s11606-009-1238-8 PMC2869410

[12] Kadmon G Pinchover M Weissbach A Case not closed: prescription errors 12 years after computerized physician order entry implementation. J Pediatr. 2017; 190:236–240.e2 29144250 10.1016/j.jpeds.2017.08.013

[13] Johnson DP Arnold DH Gay JC Implementation and improvement of pediatric asthma guideline improves hospital-based care. Pediatrics. 2018; 141 2 e20171630 29367203 10.1542/peds.2017-1630

[14] Kasmire KE Hoppa EC Patel PP Reducing invasive care for low-risk febrile infants through implementation of a clinical pathway. Pediatrics. 2019; 143 3 e20181610 30728272 10.1542/peds.2018-1610

[15] Mohan S Nandi D Stephens P Implementation of a clinical pathway for chest pain in a pediatric emergency department. Pediatr Emerg Care. 2018; 34:778–782 27649041 10.1097/PEC.0000000000000861 PMC5359077

[16] Wolff M Schinasi DA Lavelle J Management of neonates with hyperbilirubinemia: improving timeliness of care using a clinical pathway. Pediatrics. 2012; 130:e1688–e1694 23147974 10.1542/peds.2012-1156

[17] Murray AL Alpern E Lavelle J Clinical pathway effectiveness: febrile young infant clinical pathway in a pediatric emergency department. Pediatr Emerg Care. 2017; 33:e33–e37 28072664 10.1097/PEC.0000000000000960

[18] Poole NM Kronman MP Rutman L Improving antibiotic prescribing for children with urinary tract infection in emergency and urgent care settings. Pediatr Emerg Care. 2020; 36:e332–e339 29298246 10.1097/PEC.0000000000001342

[19] Benneyan JC Lloyd RC Plsek PE Statistical process control as a tool for research and healthcare improvement. Qual Saf Health Care. 2003; 12:458–464 14645763 10.1136/qhc.12.6.458 PMC1758030

[20] Kullar R Goff DA Schulz LT The “epic” challenge of optimizing antimicrobial stewardship: the role of electronic medical records and technology. Clin Infect Dis. 2013; 57:1005–1013 23667260 10.1093/cid/cit318

[21] Litvin CB Ornstein SM Wessell AM Use of an electronic health record clinical decision support tool to improve antibiotic prescribing for acute respiratory infections: the ABX-TRIP study. J Gen Intern Med. 2013; 28:810–816 23117955 10.1007/s11606-012-2267-2 PMC3663943

[22] Forrest GN Van Schooneveld TC Kullar R Use of electronic health records and clinical decision support systems for antimicrobial stewardship. Clin Infect Dis. 2014; 59 Suppl 3 S122–S133 25261539 10.1093/cid/ciu565

[23] Kimberlin DW Red Book: 2018-2021 Report of the Committee on Infectious Diseases. 2018 American Academy of Pediatrics

[24] Stevens DL Bisno AL Chambers HF; Infectious Diseases Society of America. Practice guidelines for the diagnosis and management of skin and soft tissue infections: 2014 update by the Infectious Diseases Society of America. Clin Infect Dis. 2014; 59:e10–e52 24973422 10.1093/cid/ciu444

[25] Roberts KB; Subcommittee on Urinary Tract Infection SCoQI, Management. Urinary tract infection: clinical practice guideline for the diagnosis and management of the initial UTI in febrile infants and children 2 to 24 months. Pediatrics. 2011; 128 3 595–610 21873693 10.1542/peds.2011-1330

