# Supplementary figures and images for: Reducing Antibiotic Prescription Errors in the Emergency Department: A Quality Improvement Initiative
Source: Pediatr Qual Saf. 2020 Jun 26;5(4):e314. doi: 10.1097/pq9.0000000000000314 (PMC7339249; doi:10.1097/pq9.0000000000000314)

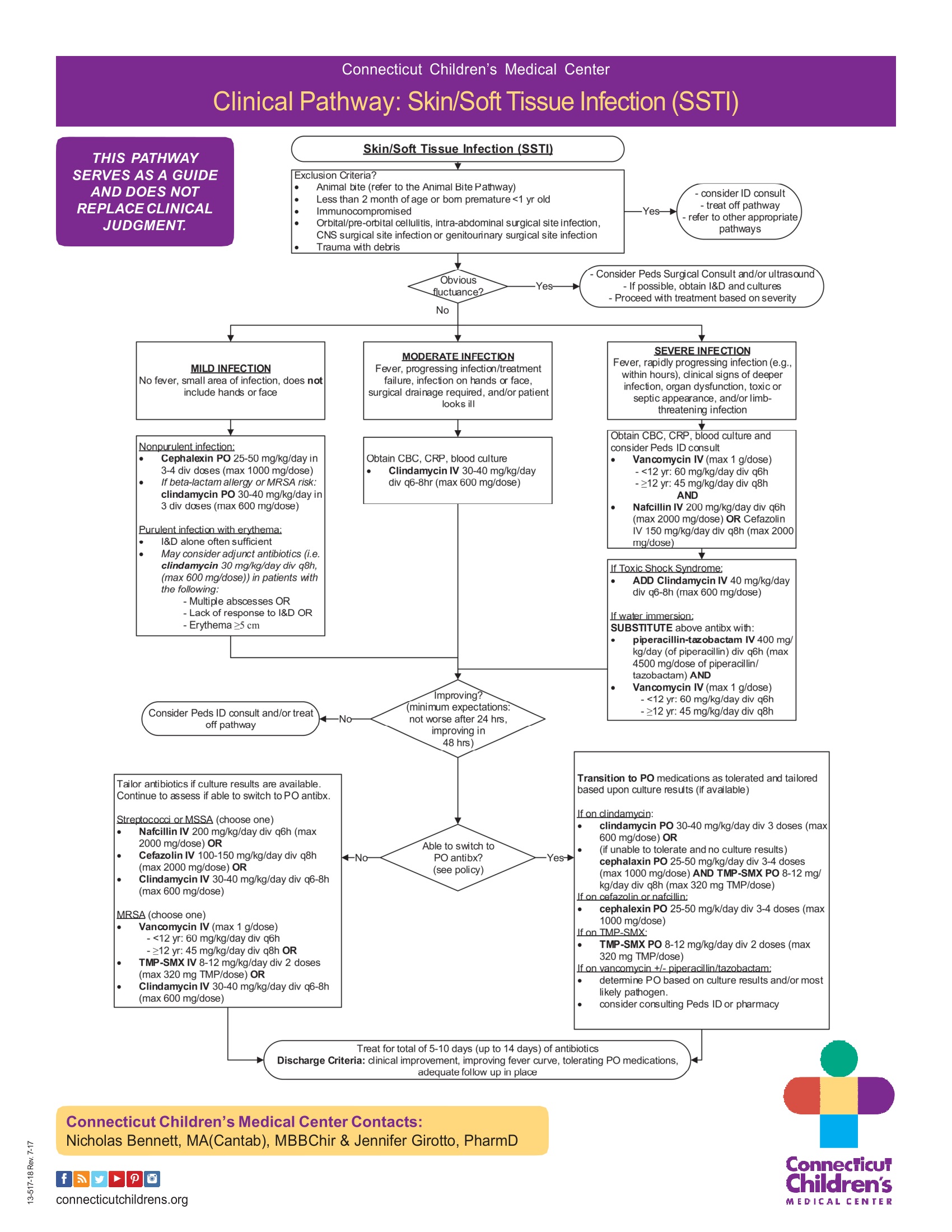

Supplement: Supplementary file 1 [file pqs-5-e314-s001.jpg]

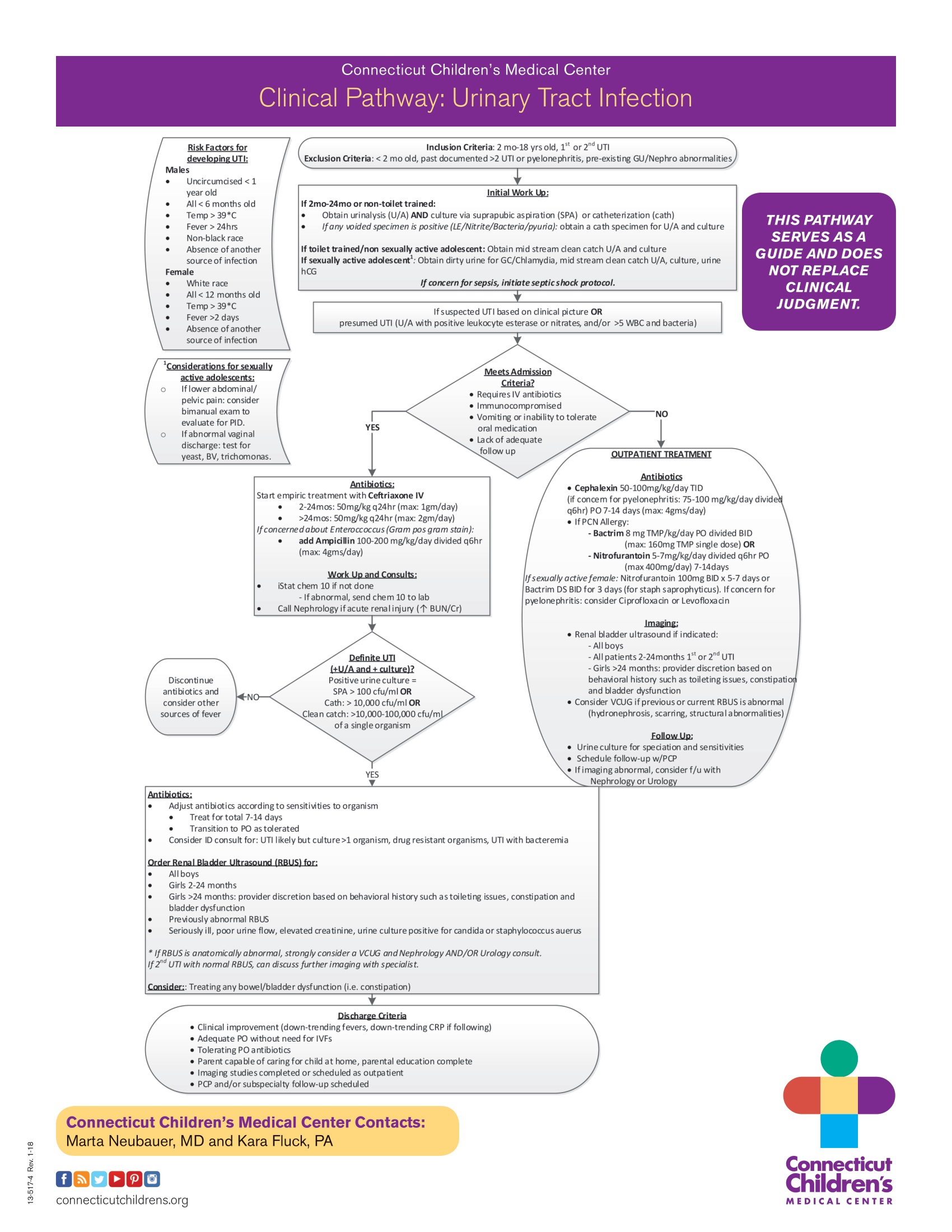

Supplement: Supplementary file 2 [file pqs-5-e314-s002.jpg]
